# Evaluation of Physico-Chemical Characteristics of Cement Superplasticizer Based on Polymelamine Sulphonate

**DOI:** 10.3390/ma17091940

**Published:** 2024-04-23

**Authors:** Asta Judžentienė, Agnė Zdaniauskienė, Ilja Ignatjev, Rūta Druteikienė

**Affiliations:** 1 Department of Organic Chemistry, Center for Physical Sciences and Technology, Saulėtekio Avenue 3, 10257 Vilnius, Lithuania; agne.zdaniauskiene@ftmc.lt (A.Z.); ilja.ignatjev@ftmc.lt (I.I.); 2 Department of Nuclear Research, Center for Physical Sciences and Technology, Savanorių Avenue 231, 02300 Vilnius, Lithuania; ruta.druteikiene@ftmc.lt

**Keywords:** polymelamine sulphonate superplasticizer, scanning electron microscopy (SEM), Brunauer–Emmett–Teller method (BET), thermo-gravimetric analysis (TGA), wavelength-dispersive X-ray fluorescence spectroscopy (WD-XRF), Raman spectroscopy

## Abstract

Cementitious materials are used to construct an engineered barrier in repositories for radioactive waste. The cement matrix may contain a variety of organic compounds, some of which are polymeric admixtures used as plasticizers. Superplasticizers (SPs) are highly effective organic cement additives for reducing water amount, increasing workability, homogeneity, plasticity and the non-segregation of mortars and grouts, improving mechanical properties and resistance to destructive environments. SPs in cement could have an impact on the long-term safety of the disposals of radioactive waste. These organic agents can leach from the cementitious matrix into groundwater and may affect the migration behaviour of radionuclides. The detailed chemical composition and other characteristics of the cement (CEM I 42.5 R, Sweden) used for the leaching experiments were evaluated. It contained mainly CaO (52.51 ± 1.37, %), and the surface area of the cement particles was 13.2 ± 1.3 m^2^/g. An insignificant increase in pH (from 12.6 ± 0.1 to 12.8 ± 0.1) was observed for the leachates over 10 days. A commercially available cement superplasticizer based on polymelamine sulphonate (PMS) Peramin SMF10 (Peramin AB, Sweden) was chosen for the research. The product’s chemical composition was analysed using wavelength-dispersive X-ray fluorescence (WD-XRF) spectroscopy, while other physico-chemical properties of the PMS superplasticizer were assessed by Raman spectroscopy and thermo-gravimetric analysis. In aqueous solutions and powders of PMS, the same most intensive features were observed at 774 cm^−1^ (ring out-of-plane deformation), 977 cm^−1^ (C-N-C bending, SO stretching) and 1055 cm^−1^ (C-N=C bending) in the Raman spectra. At up to 270 °C, the polymer was thermally stable. Raman and UV/Vis spectroscopies were used to assess the rate of the alkaline degradation of PMS superplasticizer in different aqueous solutions. No changes were observed in the hydrolytic solutions with any of the above analytical methods over a period of 3 years. The results obtained revealed a good thermal and chemical stability (in highly alkaline media, pH = 9.9–12.9) of the PMS polymer.

## 1. Introduction

To dispose of radioactive waste, such as a long-lived high- (i.e., spent nuclear fuel) and intermediate-level waste, a number of management options (waste-treatment facilities, storage facilities and deep geological repositories) are used in many countries. In Lithuania, all radioactive waste is stored near the Ignalina nuclear power plant and in the closed radioactive-waste storage facility (in the Širvintos district). Cementitious materials containing a common Portland cement, a shotcrete-type cement mixture consisting of a CEM I-type cement (pure Portland cement with more than a 95% clinker content) and silica fume, and a low-alkaline cement mix comprised of a CEM III (usually about 45% cement and more than 40% blast-furnace slag) and nanosilica, are applied for the building of the engineered barrier of the radioactive-waste repositories [1].

One of the most commonly used techniques prior to near-surface or underground disposal is the solidification of low- and intermediate-level radioactive waste with cementitious materials [2,3,4,5]. In assessing the long-term safety of radioactive waste repositories, the interaction between radionuclides and cement-based barrier materials is of great interest [6,7,8,9,10]. The interplay is dependent on both the nature of the radionuclides and the type of cement [10,11,12,13,14]. The retention or migration of radionuclides from the disposal facility is related to several factors specific to a cementitious backfill, including a significant pH gradient, temperature, electrolyte composition, presence of organic compounds, redox potential and ionic strength [6,8,9,10,14].

Depending on the type of waste, the cement matrix of radioactive-waste packages may contain various organic substances: some are polymeric additives, such as (super)plasticizers, while others are hydrolysed and radio-oxidized polymers formed during the operation of nuclear-fuel cycling facilities. 

It is well established that hyper-alkaline conditions in cementitious media can cause the ageing and degradation of polymeric additives. This can result in the formation of new organic compounds with altered chemical properties [15]. 

Concrete admixtures are usually made up of one or more polymeric organic compounds. Superplasticizers (SPs) are additives known as “high-range water-reducing admixtures”; they can be used at much higher doses than conventional plasticizers. SPs are highly efficient organic substances that can be used as additives to decrease the amount of water and to ensure the homogeneity and non-segregation of mortars and grouts. Additionally, they can reduce porosity and improve mechanical strength, workability and resistance to destructive environments. SPs are able to provide the desired fluidity and plasticity of cement without causing undesirable effects [16]. SPs are high-molecular-weight polymers that are soluble in water [16,17,18,19,20]. The ability of SPs to reduce the amount of water by 12–25% without affecting their workability results in the production of high-strength concrete with a lower permeability [19,21,22]. 

Currently, the key organic compounds utilised include sulphonated naphthalene-formaldehyde condensates, modified lignosulphonates, sulphonated melamine-formaldehyde condensates and polycarboxylate-ether-based molecules [1,23,24,25,26]. Lignin is a first-generation water-reducing SP, based on natural sources, which is a byproduct of pulp production [24,25]. Polynaphthalene sulphonate or polymelamine sulphonate is a second-generation water-reducing SP, based on synthetic polymers of naphthalene or melamine. Polycarboxylate is a third-generation high-range water-reducing SP, based on a synthetic polycarboxylate copolymer which has a comb-like microstructure [24,25,26]. Solubility is achieved by the presence of adequate sulphonate, carboxylic or hydroxyl groups attached to the main organic repeating unit, which is usually anionic [1,16,22]. Sulfonated SPs induce a negative charge on cement particles, dispersing them by electrostatic repulsion, whereas with the polycarboxylate-based polymer, the dispersion mechanism is mainly controlled by steric hindrance [27,28].

According to market analysts [29], the sulphonated melamine-formaldehyde (PMS) condensates segment accounts for up to 31% of the total market and is the highest share of any type of concrete SP. PMS has been widely used in cement, dry mixtures of mortars and gypsum compositions. Also, PMS is used as a plasticizer in plaster formulations with retarding, accelerating or air-entrainment effects. Air lime-based injection grouts containing polymeric SPs (including PMS) can be used for historic masonry repairs [20]. PMS, keeping a very low free formaldehyde quantity, can be applied in the leather industry (for re-tanning) [30]. In the above study, minor amounts (1–3%) of the polymer were used for leather processing that provided about 98% of the exhaustion of dyes.

According to the hypothesis that the migration behaviour of radionuclides in concrete systems of repositories for long-term radioactive waste can be influenced by the degradation of SPs over a long period of time with the supply of relatively low-molecular-weight polymers and their release into groundwater, it is necessary to investigate this phenomenon. Furthermore, to the best of our knowledge, there is a definitive lack of information on the potential products of PMS degradation/hydrolysis. 

The main objectives of this research are (i) to analyse the detailed chemical composition and physical properties of the Portland cement (CEM I-type) used in the leaching experiments; (ii) to examine the chemical and physical characteristics of a commercially available cement superplasticizer made from polymelamine sulphonate (PMS); and (iii) to assess the rate of the long-term alkaline degradation of the PMS superplasticizer in various aqueous solutions (with pH levels up to 13) by conducting spectroscopic analysis (using Raman and UV/Vis techniques) of the PMS hydrolytic solutions.

## 2. Materials and Methods

### 2.1. Compositional Analysis of Cement 

Commercially available Portland cement (CEM I 42.5 R, Heidelberg Cement Group, Stocholm, Sweden) was used for the tests. The chemical composition of the cement was examined using a fluorescent X-ray spectrometer with wave dispersion Axios mAX (PANanalytical, Almelo, The Netherlands) and a Carbon and Sulphur analyser CS-2000 (Eltra, Haan, Germany).

### 2.2. Preparation of Concrete Samples

Thin plates (150 mm × 100 mm × ~3 mm) of concrete were prepared with a low amount of tap water (1.2:1, *v*/*v*, cement/H_2_O) for the leaching experiments. The slabs containing CEM I 42.5 R and 2% PMS were hardened for 20 days. The solutions for leaching experiments were prepared with a low amount of deionized tap and moderately saline (0.5% NaCl) water.

### 2.3. BET Analysis

The specific surface area of the cement samples and total pore volume were measured by means of N_2_ physisorption at −196 °C (Micromeritics TriStar II 3020 V1.03 Micromeritics Instrument Corp., Norcross, GA, USA). Before analysis, the samples were outgassed under a vacuum at 100 °C for 4 h. The total pore volume was measured from the adsorption isotherm by the nitrogen uptake at a relative pressure of p/p_0_ = 0.99. The specific surface area was calculated using the BET (Brunauer–Emmett–Teller) method. The pore distribution and the average pore width were examined by Barrett–Joyner–Halenda (BJH) analysis.

### 2.4. Scanning Electron Microscopy (SEM)

The surface morphology was examined in a dual-beam system FE-SEM-FIB Helios Nanolab 650 (FEI Company, Hisboro, OR, USA), using 2–5 kV accelerating voltage. Different magnification levels (up to 20,000) were chosen for this analysis, and the magnification of 650 was shown as the most representative. The slabs (150 mm × 100 mm × ~3 mm) were hardened for 90 days.

### 2.5. Chemical Analysis by Wavelength-Dispersive X-ray Fluorescence (WD-XRF) Spectroscopic Method

A detailed chemical analysis of the PMS product (Peramin SMF10) was carried out using the WD-XRF spectroscopic method, using an AxiosmAX (Malvern PANalytical, Malvern, Worcestershire, UK) spectrometer equipped with an SST-mAX X-ray tube (Rh anode, 4 kW), and the results of the quantitative analysis were obtained using the Omnian XRF software (PANalytical, Almelo, The Netherlands, https://www.malvernpanalytical.com/en/products/category/software/x-ray-fluorescence-software/omnian (accessed on 6 March 2024)).

### 2.6. Thermo-Gravimetric Analysis (TGA)

The physico-chemical properties of the PMS superplasticizer were evaluated by TGA, using a Perkin Elmer Inc. STA6000 thermal analyser (Waltham, MA, USA) in air up to 900 °C at 5–10 °C min.

### 2.7. Preparation of Aqueous Solutions of Superplasticizer PMS

Deionized water was obtained by the Direct-Q ^®^3UVwater-Purification system (Type 1) under 20.0 ± 1.0 °C and 18.2 MΩ-cm conditions. Solutions of PMS (4 and 10%) were prepared in bi-distilled, deionized, tap and synthetic saline water (0.5% NaCl). The pH values (9.90, 12.41, 12.43 and 12.88) of solutions were achieved using 0.5 M KOH.

### 2.8. Analysis of PMS Hydrolytic Solutions by Spectroscopic (Raman and UV/Vis) Techniques

A Lambda 25 UV/Vis (Perkin Elmer, Buckinghamshire, UK) spectrometer was used for the spectrophotometric analysis (range 200–1100 nm) of various PMS hydrolytic samples. RamanFlex 400 (Perkin Elmer Inc., Shelton, CT, USA) was equipped with a thermoelectrically cooled (−50 °C) CCD camera and a fibre-optic cable for the excitation and collection of the Raman spectra. The 785 nm beam of the diode laser was used as the excitation source. The laser power on the samples was set at 100 mW. The integration time was 300 s.

The time periods for analyses were 0, 3, 7, 15, 21, 28 and 45 days, and then the frequency lowered to 6, 8, 10 and 12 weeks, to up to 3 years.

### 2.9. Statistical Analysis

The obtained data were presented in range intervals, by means and standard deviation (SD) values, using XLSTAT (trial version, Addinsoft 2014, Paris, France).

## 3. Results and Discussion

### 3.1. The Chemical and Physical Characteristics of a Commercial Cement Superplasticizer Based on Polymelamine Sulphonate

**Figure 1:**
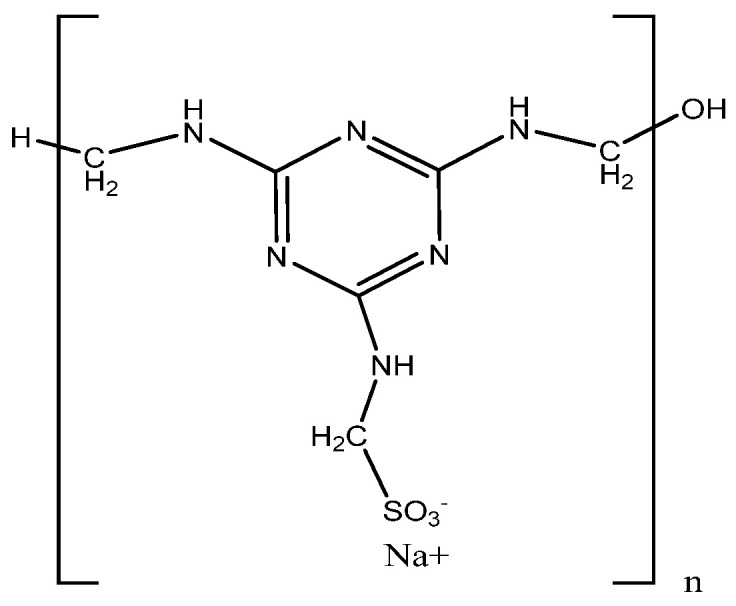
Chemical structure of polymelamine sulphonate was drawn using the ChemDraw (version 16.0) molecule editor.

PMS condensates are artificial polymers characterised by a structural formula in which each repeating unit contains a single sulphonate group (Figure 1). It is known that the condensation number (n) often falls within the range of 50 to 60, and a molecular weight could vary from 12,000 to 15,000 [16]. The synthesis of PMS takes place in two stages. The first step involves a condensation reaction between melamine, sulphite and formaldehyde. Under alkaline conditions, formaldehydes react with the amino groups of melamine (2,4,6-triamino-s-triazine), producing a methylated melamine. Sodium bisulphite sulphonates one of the methylol groups under the same alkaline conditions. In the second stage, poly-condensation is performed under acidic conditions [23,25,31].

The commercially available product under the name Peramin ^®^ SMF10 (Peramin AB, Upplands Väsby, Sweden) is based on polymelamine sulphonate (PMS). The product is an off-white powder or a dust with a density of 450–650 kg/m^3^, a pH of 5%, an aqueous solution of approximately 8.8 and a dosage of 0.2–2.0% by weight. The main functions of the SP are to reduce water content, to give homogeneity, to lower porosity and to not segregate mortars and grouts, thus increasing the mechanical strength, fluidity and workability. In addition, the product can be used to improve the resistance of the cement to aggressive environments.

### 3.2. Chemical and Physical Characteristics of Commercial Cement

Commercially available Portland cement (CEM I 42.5 R, Heidelberg Cement Group, Stocholm, Sweden) was used for the experiments, as cement CEM I and CEM III are commonly used for the construction of radioactive-waste repositories.

**Figure 2:**
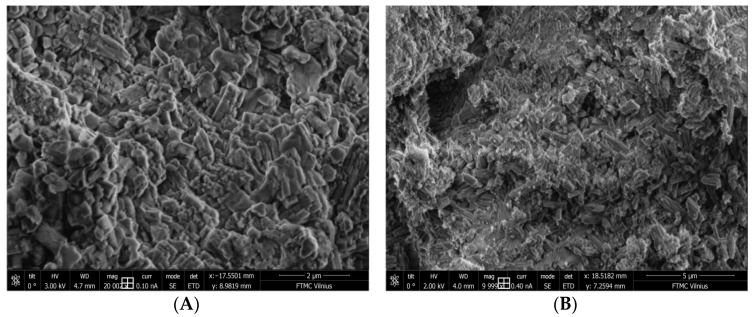
Selected SEM pictures of cement CEM I 42.5 R grains with different magnifications: (**A**)—20,000 and (**B**)—10,000.

In many cases, the composition of concrete admixtures is not fully known for commercial reasons. Ordinary Portland cement consists mainly of the following clinker minerals (95–100%): calcium silicates (Ca_3_SiO_3_ and Ca_2_SiO_4_), tricalcium aluminate (Ca_3_Al_2_O_6_) and tricalcium alumino ferrite (Ca_4_(Al_x_Fe_(1−x)4_O_10_). There might be a small quantity of other minerals present, including CaCO_3_, alkali sulphates, gypsum and various oxides [32]. Typical scanning electron microscopy (SEM) images of cement are shown in Figure 2.

The chemical composition of cement is mostly based on Na^+^, K^+^ and Ca^2^+. As a result, it creates a highly alkaline medium with a pH level that can reach up to 13 over a long period of time. The gradual changes in pore water composition, from typical young-stage cement leachates with a pH above 13 to leachates with a pH below 10, caused the degradation of the hydrated cement and the subsequent formation of new phases, such as calcium silicate hydrate (C-H-S) [33,34,35,36].

The BET (Brunauer–Emmett–Teller method) surface area of the cement particles, calculated on the basis of the amount of adsorbed nitrogen gas monolayer, was determined to be ~13.2 m^2^/g (Table 1). The analysis of the pore surface-area distribution revealed that the majority of the cement pores could be attributed to the micro-pores within the size range of 1–10 nm.

The main components of cement obtained by X-ray fluorescence spectroscopy are given in Table 2.

Cement is used widely in waste repositories due to its ability to buffer a high pH value of water, to maintain many radionuclides as low soluble hydroxides, to lead a substrate for sorption, and thus to retard the migration of long-lived radionuclides [37,38,39,40]. An important feature of cement systems is the formation of high-pH conditions [41]. This occurs as the result of hydration reactions as cement is mixed with water, principally forming calcium-silicate-hydrates (CSH) of different compositions and portlandite (Ca(OH)_2_) [33,35,39,40,41]. The dissolution of these phases over time leads to the development of higher pH values.

As a result of cement degradation, four stages of pH evolution in the hydrated cement pore fluid were observed at a temperature of 25 °C [34]: (I) an initial period of very a high pH value (>12.5) is established due to the dissolution of soluble salts, such as KOH and NaOH; (II) a period when pH is buffered at 12.5 by the dissolution of Ca(OH)_2_; (III) a stage (12.5 > pH > 10) that is is more complex and is controlled by the dissolution of CSH, where Ca is preferentially leached from the solid phase until the Ca:Si ratio in the CSH is the same as that in solution, after which the congruent dissolution of CSH occurs; (IV) and after the depletion of CSH, the pH of the pore water is controlled by secondary minerals, such as calcite (pH = incoming water).

The pH level is a critical factor in monitoring the health of concrete [42]. Three types of water (deionized, tap and moderately saline water containing 0.5% NaCl) were used for leaching experiments over 10 days at room temperature (20 ± 2 °C). An insignificant increase in pH from 12.6 ± 0.1 up to 12.8 ± 0.1 was observed for all leachates, and it was highest for solutions prepared with deionized water. The increase could be explained by the elution of cations of Ca, Na, K and OH^−^ from the cement matrix. Similar tendencies (the pH of the fresh concrete was ≈ 13.1 and increased gradually up to 13.8) were observed in concrete throughout the hydration procedure over 90 days [42].

### 3.3. Characterization of Cementitious Material by Scanning Electron Microscopy (SEM)

**Figure 3:**
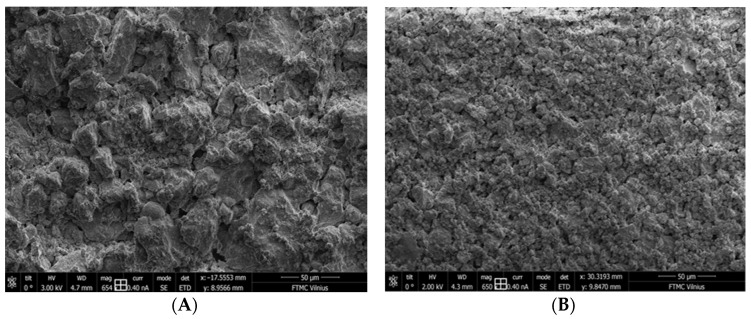
SEM images of concrete before leaching; magnification 650 times. (**A**)—image of cement CEM I 42.5 R sample (without superplasticizer); (**B**)—image of cement CEM I 42.5 R + 2% PMS sample prepared in a low amount of tap water (1.2:1, Vol. cement: Vol. H_2_O).

The surface morphology was assessed in a dual-beam FE-SEM-FIB system. Different magnification levels were chosen for this analysis, and the magnification of 650 was shown as the most representative. Thin plates were hardened for 90 days. SPs could improve the physical and, accordingly, mechanical properties of concrete. Figure 3 demonstrates the dispersibility effects of PMS. It can be seen that sample B is more homogeneous in terms of size and shape in the SEM image due to the admixture.

### 3.4. Chemical Composition of Peramin ^®^ SMF10

A detailed chemical analysis of the product was carried out by the wavelength-dispersive X-ray fluorescence (WD-XRF) spectroscopic method. A major constituent was found: a PMS ((C_3_N_3_(NH)_3_(CH_2_)_3_SO_3_Na)_n_ + H_2_O) polymer in a quantity of over 99.8%. In addition, nine minor constituents, Al_2_O_3_, P_2_O_5_, K_2_O, ZnO, Na_2_O, SiO_2_, CaO, Cl^−^ and Fe_2_O_3_, were identified (Table 3).

The chemical composition (%) of the product Peramin ^®^ SMF10 according to the Product Data Sheet is the following: dry content 93.2 (requirement is 93.5 ± 2.0), Na_2_O 0.658, K_2_O 12.0 (limit is <13) and Cl^−^ (as ions) 0.004 (requirement is <0.05).

It should be noted that the use of PMS may be restricted due to the potential presence of free formaldehyde in the solution, which has been classified as a human carcinogen since 1981 [43].

### 3.5. Raman Spectroscopic Analysis of Polymelamine Sulphonate (PMS) Powder (Commercial Product Peramin SMF 10)

**Figure 4:**
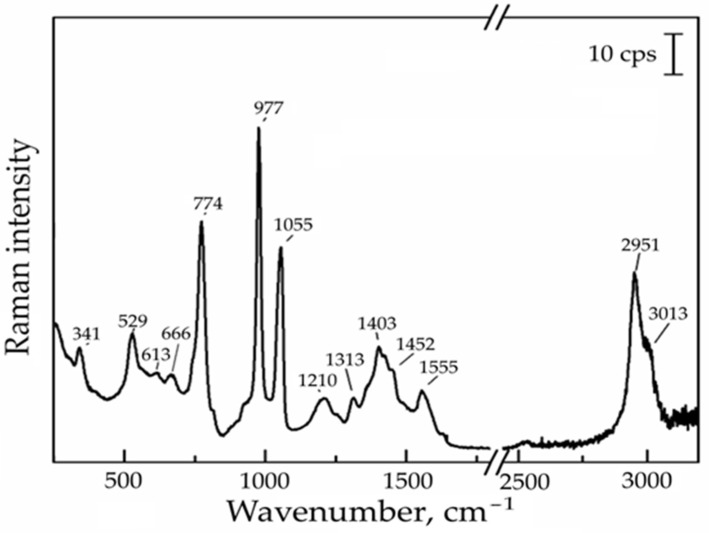
Raman spectrum of polymelamine sulphonate (PMS) powder (commercial product Peramin SMF 10).

As far as we are aware, there is no Raman spectrum of PMS available in the scientific literature. The main features of PMS were observed at 341 cm^−1^ (H-N-C-N twisting), 529 cm^−1^ (N-H twisting), 613 cm^−1^ (ring in-plane deformation), 666 cm^−1^ (ring breathing), 774 cm^−1^ (ring out-of-plane deformation), 977 cm^−1^ (C-N-C bending, SO stretching), 1055 cm^−1^ (C-N=C bending), 1210 cm^−1^ (NH rocking, C-N stretching), 1313 cm^−1^ (ring deformation, C-N asymmetric stretching), 1403 cm^−1^ (C-N stretching, C-N=C ring symmetric deformation, N-H in-plane bending), 1452 cm^−1^ (CH_2_ deformation), 1555 cm^−1^ (ring deformation bending), 2951 cm^−1^ and 3013 cm^−1^ (C-H stretching) in the Raman spectrum (Figure 4), which were in agreement with the literature [44,45].

### 3.6. Thermo-Gravimetric Analysis (TGA) of Peramin ^®^ SMF10

TGA was performed at a temperature range of 30–800 °C in the air. The thermal stability of the PMS polymer remained up to 270 °C. The residual ash of the sample may consist of finely divided SiO_2_ or metal carbonates or oxides (according to the composition from the elemental analysis (Table 3)).

**Figure 5:**
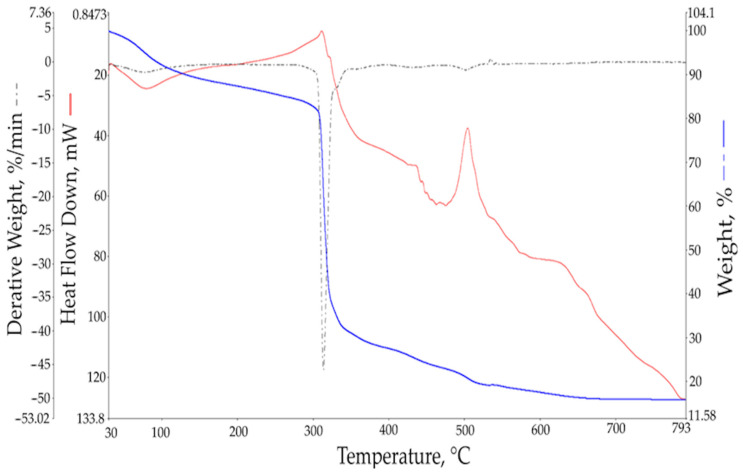
Thermo-gravimetric analysis of Peramin SMF10 (based on PMS). Curves: blue—percentage weight loss (TG); dashed black—derivative weight loss (DTG) (%/min) and red—differential scanning calorimetry (DSC) (heat flow (mW)).

Three mass losses were observed (Figure 5). The following thermal processes were revealed: (1) the 36–136 °C endothermic evaporation of adsorbed H_2_O (approx. 7% mass loss); (2) the 284–349 °C exothermic decomposition of the polymer/polymer matrix, possibly with the formation of monomers and the further oxidation of monomers (48% mass loss after the evaporation of adsorbed H_2_O); (3) and the 473–546 °C exothermic complete oxidation of de-polymerization products (final mass, 15% of the initial sample weight).

### 3.7. Analysis of PMS Hydrolytic Solutions

In general, concrete additives may have an impact on the long-term safe disposal of radioactive waste in several ways: (i) if these admixtures degrade over time and their degradation products, such as CO_2_, interact with the cement matrix, they can alter the mineral composition of the cement; (ii) if they act as complexing ligands, they may impact the mobility of radionuclides within the engineered barrier; and (iii) they can cause changes in the chemical-boundary conditions of the system, like pH or Eh. SPs have the ability to interact with the surface of cement particles, which can change the affinity of the particles towards radionuclides. The presence of an organic coating on cement minerals can either support (via ternary-surface-complex formation) or inhibit (through competition) the absorption of radionuclides [33].

Spectroscopic (Raman and UV/Vis) analyses have been undertaken in order to investigate the rate of alkaline degradation of the PMS superplasticizer in various aqueous solutions at room temperature (20 ± 2 °C) under natural light in atmospheric conditions.

#### 3.7.1. Spectrophotometric Analysis of PMS Hydrolytic Samples

**Figure 6:**
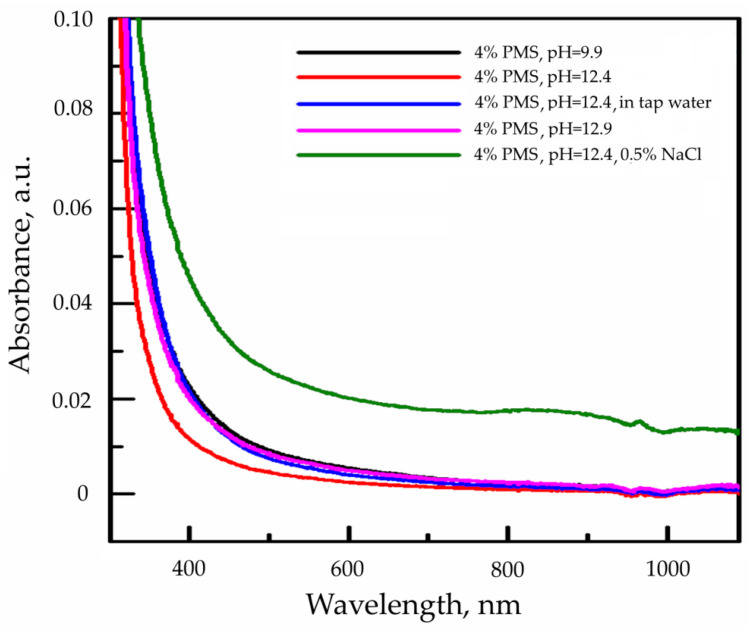
UV/Vis spectra of different polymelamine sulphonate (PMS) hydrolytic solutions (sampling time: 3 years).

A UV/Vis spectrophotometric analysis of PMS samples was performed in a range from 200 to 1100 nm. The identical curves were obtained for just-prepared PMS solutions and for samples after 3 years of hydrolysis (Figure 6).

#### 3.7.2. Raman Spectroscopic Analysis of PMS Hydrolytic Samples

**Figure 7:**
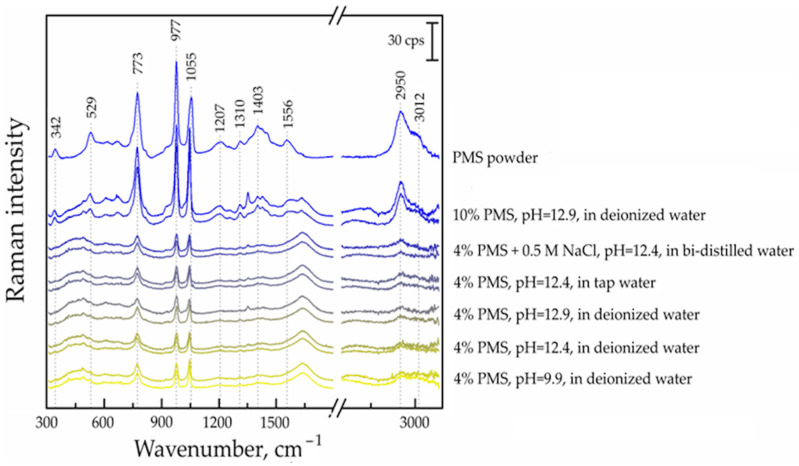
Raman spectra of different polymelamine sulphonate (PMS) hydrolytic solutions (sampling time: 1 day and 3 years).

The Raman spectra of various PMS hydrolytic solutions were recorded over time using an Echelle-type spectrometer (Perkin Elmer, Shelton, CT, USA) (Figure 7).

In the Raman spectra, the vibrational modes of the PMS of the substrate are the most observed. The intensity of the signals correlates with the amount of PMS added. The most intense vibrational modes are from the aromatic heterocycle of the PMS. The strongest signals of PMS in the solutions were identified in the Raman spectra at 774 cm^−1^ (ring out-of-plane deformation), 977 cm^−1^ (C-N-C bending, SO stretching) and 1055 cm^−1^ (C-N=C bending). These spectra are similar to the Raman spectrum of the PMS powder. The upper curves showed the Raman spectra of solutions after three years, while the lower curves represented just-made samples (Figure 7). The identical curves were obtained for the just-prepared PMS solution and for samples after 3 years of hydrolysis.

No changes were observed in the hydrolytic solutions with any of the above-mentioned spectroscopic methods of analysis. A comparison of the obtained results with the literature data is not possible due to the fact that the number of publications related to PMS hydrolysis is very limited.

## 4. Conclusions

The present study has contributed to our understanding of the application of (super)plasticizing agents as admixtures in cementitious materials for constructing radioactive-waste repositories. In general, the performed research has provided some new data for consumers, concerning the physico-chemical properties and characteristics of commercially available cement superplasticizer based on polymelamine sulphonate (Peramin SMF10) and cement (CEM I 42.5 R). The results obtained by TGA showed the good thermal stability (up to 270 °C) of the PMS polymer. This study presents new information regarding the stability of PMS in highly alkaline media. Currently, there is a definite lack of information on the potential products of PMS degradation/hydrolysis. The chemical stability of PMS polymer in hyper-alkaline aqueous solutions (pH = 9.9–12.9) was assessed by both UV/Vis and Raman spectroscopic analyses over a period of 3 years. The time period is short enough that the monitoring of the PMS hydrolysis process under highly alkaline conditions will be continued.

## Figures and Tables

**Table 1:** Physical characteristics of cement CEM I 42.5 R; values are presented as means ± SD (n = 3).

Sample Name	Area of BET Surface, m^2^/g	Area of Micro-Pores, m^2^/g	Area of External Surface, m^2^/g	Volume of Pores, cm^3^/g	Pore Diameter Range, nm	Average Pore Diameter, nm
CEM I	13.2 ± 1.3	7.1 ± 0.2	6.1 ± 0.1	0.00	2.6 ± 0.3–86.5 ± 3.3	14.6 ± 2.5

**Table 2:** Chemical composition of the raw cement (CEM I 42.5 R) material; values of constituents are expressed as means ± SD (n = 3).

No.	Compound	Percentage (Mass %)
1.	CO_2_	13.90 ± 0.18
2.	O	5.93 ± 0.04
3.	CaO	52.51 ± 1.37
4.	SiO_2_	14.98 ± 0.12
5.	Al_2_O_3_	3.19 ± 0.01
6.	SO_3_	2.98 ± 0.03
7.	Fe_2_O_3_	2.78 ± 0.05
8.	MgO	2.13 ± 0.01

**Table 3:** Full chemical composition (values were expressed as means ± SD (n = 3)) of the commercial product Peramin SMF10.

No.	Compound	Percentage (Mass %)
1.	PMS	99.824 ± 0.102
2.	P_2_O_5_	0.054 ± 0.004
3.	ZnO	0.033 ± 0.019
4.	Na_2_O	0.021 ± 0.001
5.	SiO_2_	0.021 ± 0.003
6.	Al_2_O_3_	0.017 ± 0.001
7.	CaO	0.017 ± 0.008
8.	K_2_O	0.006 ± 0.001
9.	Fe_2_O_3_	0.005 ± 0.001
10.	Cl^−^	0.004 ± 0.001

## Data Availability

Data are contained within the article.
